# Risk Factors for Campylobacteriosis of Chicken, Ruminant, and Environmental Origin: A Combined Case-Control and Source Attribution Analysis

**DOI:** 10.1371/journal.pone.0042599

**Published:** 2012-08-03

**Authors:** Lapo Mughini Gras, Joost H. Smid, Jaap A. Wagenaar, Albert G. de Boer, Arie H. Havelaar, Ingrid H. M. Friesema, Nigel P. French, Luca Busani, Wilfrid van Pelt

**Affiliations:** 1 Istituto Superiore di Sanità, Department of Veterinary Public Health and Food Safety, Rome, Italy; 2 National Institute of Public Health and the Environment (RIVM), Centre for Infectious Disease Control, Bilthoven, The Netherlands; 3 Bologna University, Department of Veterinary Medical Sciences, Bologna, Italy; 4 Central Veterinary Institute of Wageningen UR, Department of Bacteriology and TSEs, Lelystad, The Netherlands; 5 Utrecht University, Department of Infectious Diseases and Immunology, Utrecht, The Netherlands; 6 World Health Organization Collaborating Centre for Reference and Research on Campylobacter/OIE Reference Laboratory for Campylobacteriosis, Lelystad/Utrecht, The Netherlands; 7 Utrecht University, Institute for Risk Assessment Sciences, Utrecht, The Netherlands; 8 Massey University, Infectious Disease Research Centre, Palmerston North, New Zealand; Cornell University, United States of America

## Abstract

**Background:**

Campylobacteriosis contributes strongly to the disease burden of food-borne pathogens. Case-control studies are limited in attributing human infections to the different reservoirs because they can only trace back to the points of exposure, which may not point to the original reservoirs because of cross-contamination. Human *Campylobacter* infections can be attributed to specific reservoirs by estimating the extent of subtype sharing between strains from humans and reservoirs using multilocus sequence typing (MLST).

**Methodology/Principal Findings:**

We investigated risk factors for human campylobacteriosis caused by *Campylobacter* strains attributed to different reservoirs. Sequence types (STs) were determined for 696 *C. jejuni* and 41 *C. coli* strains from endemic human cases included in a case-control study. The asymmetric island model, a population genetics approach for modeling *Campylobacter* evolution and transmission, attributed these cases to four putative animal reservoirs (chicken, cattle, sheep, pig) and to the environment (water, sand, wild birds) considered as a proxy for other unidentified reservoirs. Most cases were attributed to chicken (66%) and cattle (21%), identified as the main reservoirs in The Netherlands. Consuming chicken was a risk factor for campylobacteriosis caused by chicken-associated STs, whereas consuming beef and pork were protective. Risk factors for campylobacteriosis caused by ruminant-associated STs were contact with animals, barbecuing in non-urban areas, consumption of tripe, and never/seldom chicken consumption. Consuming game and swimming in a domestic swimming pool during springtime were risk factors for campylobacteriosis caused by environment-associated STs. Infections with chicken- and ruminant-associated STs were only partially explained by food-borne transmission; direct contact and environmental pathways were also important.

**Conclusion/Significance:**

This is the first case-control study in which risk factors for campylobacteriosis are investigated in relation to the attributed reservoirs based on MLST profiles. Combining epidemiological and source attribution data improved campylobacteriosis risk factor identification and characterization, generated hypotheses, and showed that genotype-based source attribution is epidemiologically sensible.

## Introduction

Virtually all people in The Netherlands (∼16 million population) possess serological evidence of multiple exposures to *Campylobacter* spp. during the course of their lives, although most infections pass with no, or mild, symptoms [1]. With an estimated 90000 symptomatic infections occurring annually, campylobacteriosis is the most frequent cause of acute bacterial gastroenteritis in The Netherlands [2]–[4]. In 2010, the incidence of laboratory-confirmed campylobacteriosis was 50 per 100,000 inhabitants, the highest ever recorded in the Dutch population since 1996. Up to 88% of these infections were acquired domestically. Hospitalization was required in approximately a quarter of laboratory-confirmed cases [5]. Most infections occur sporadically, with outbreak-related cases representing less than one percent of the total number of *Campylobacter* infections [6].

Apart from acute gastroenteritis, campylobacteriosis may lead to more severe, occasionally long-term, sequelae, such as Guillain-Barré syndrome, reactive arthritis, and irritable bowel syndrome [7], [8], causing considerable morbidity and economic impact on the Dutch population [2], [4], [8]. *Campylobacter* spp. are commensally widespread in the intestines of wild and domesticated animals, resulting in contamination of the environment, including water sources [9]. Although *Campylobacter* spp. are mostly perceived as food-borne pathogens, there is evidence for other transmission pathways, including direct and indirect contact with infectious animals, people, and environments [10]–[13].

Evidence of host-adapted *Campylobacter* strains exists [14]. However, the relative importance of each reservoir in zoonotic transmission remains unclear. Novel host-associated adaptive mutation and recombination events are frequent in *Campylobacter* spp., resulting in populations that are not strongly structured into differentiated clusters; thus, predicting host from genotype is challenging [14].

Several case-control studies have evidenced that consumption of chicken is an important risk factor for human campylobacteriosis [10]–[13], [15]. Poultry and avian species in general are the preferential host for *Campylobacter* spp., and during processing retail poultry carcasses may become contaminated [6], [9], [16], [17]. As *Campylobacter* strains of chicken origin may reach humans through pathways other than food [18], the consumption and handling of chicken may account for up to 40% of human infections, while up to 80% may be attributed to the chicken reservoir as a whole [9].

Case-control studies are insufficient for attributing human infections to the different reservoirs because they can only trace back to the points of exposure (e.g. food items consumed), which may not point to the original (amplifying) reservoirs because of cross-contamination. Attributing human infections to specific reservoirs is crucial to prioritize, implement, and measure the impact of targeted interventions [19]. Human *Campylobacter* infections can be attributed to specific reservoirs by estimating the extent of subtype sharing between strains isolated from humans and reservoirs [19]. Multilocus sequence typing (MLST) [20], [21] is a typing methodology that is widely used internationally for this purpose [14], [21]–[25]. MLST allows for the identification of genetic lineages in *Campylobacter* populations by indexing the variation present in seven housekeeping genes. A unique sequence pattern is assigned to a sequence type (ST), while closely related STs sharing the same alleles at different loci are considered as belonging to the same clonal complex (CC), the members of which possess a common ancestor [20]. Several modeling approaches can then be applied to MLST data to attribute *Campylobacter* strains from human cases to different reservoirs, e.g. [23].

With a focus on The Netherlands, the aims of this study were: 1) to attribute human *Campylobacter* infections to four putative animal reservoirs (chicken, cattle, sheep, and pig) and to the environment; 2) to combine the available case-control data [10] with the results of the attribution analysis to explore risk factors at the point of exposure for human campylobacteriosis caused by strains highly associated with the different reservoirs.

## Materials and Methods

### Human data

We used *Campylobacter* data from the so-called CaSa study, a large case-control study on risk factors for sporadic salmonellosis and campylobacteriosis conducted in The Netherlands between April 2002 and April 2003. A detailed description of the methodology and results of the CaSa study is available elsewhere [10], [26].

A total of 2858 *C. jejuni* and 257 *C. coli* cases were identified by the Dutch Regional Public Health Laboratories (RPHL) and assigned to species using molecular methods [27], [28] at the Central Veterinary Institute (CVI) in Lelystad, The Netherlands. Cases were interviewed by means of a questionnaire sent by the RPHL together with the laboratory test results to the prescribing physician who forwarded these to the corresponding patient. After exclusion of cases who: 1) did not return or complete successfully the questionnaire (1679 cases); and 2) had a recent or unknown history of foreign travel, and/or lived outside The Netherlands (338 cases), 1019 *C. jejuni* and 79 *C. coli* cases were enrolled in the study.

Based on historic surveillance data of the number of *Campylobacter* and *Salmonella* infections in the RPHL service areas, the expected numbers of cases by age (0–4, 5–17, 18–29, 30–44, 45–59, and ≥60 years), sex, degree of urbanization (urban: >2500 addresses/km^2^; urbanized: 500–2500 addresses/km^2^; rural: <500 addresses/km^2^), and season (April–June 2002, July–September 2002, October–December 2002, January–April 2003) were obtained. Controls were randomly selected from population registries within the RPHL service areas by frequency matching (aiming at two controls per case) according to the expected number of cases by age, sex, degree of urbanization, and season. A total of 10250 controls were approached in anticipation of an expected response rate of 25%. Of these, 3409 (33%) controls returned the postal questionnaire. After exclusion of controls who: 1) had travelled abroad (244 controls);or 2) did not provide reliable information (46 controls), 3119 controls were enrolled in this study.

Cases and controls were asked to fill in the aforementioned questionnaire to collect information regarding food consumption, kitchen hygiene, food processing, contact with animals, occupational exposure, history of travel, recreational water activity, medication use, history of chronic diseases, and contact with people with gastroenteritis. Questions covered the 7 days prior to symptoms onset (cases) or completion of the questionnaire (controls). Parents were asked to complete the questionnaire on behalf of their children. Missing values were handled using multiple imputation [29].

Isolates from 980 cases (919 *C. jejuni* and 61 *C. coli*) identified by the RPHL were successfully typed with MLST as described elsewhere [20], [21]. Of these, 737 cases (696 *C. jejuni* and 41 *C. coli*) were cases enrolled in the study, as the other 243 typed cases were not eligible for enrollment because they had travelled abroad or did not return/complete successfully the questionnaire. Purification and sequencing of PCR products were done by Macrogen Inc, Korea. The software Bionumerics 5.10 was used to analyze sequence data.

Differences in relative frequencies of the five most frequently reported STs (ST-53, ST-50, ST-21, ST-48, and ST-45) and CCs (CC-21, CC-45, CC-206, CC-257, and CC-48) were examined for the variables age, sex, degree of urbanization, and season, using Pearson's χ^2^ test (α-level: 0.05).

### Animal and environmental data

As only few Dutch *Campylobacter* reference strains typed with MLST were available for the animal reservoirs (232 strains) and for the environment (106 strains) [30] (Table 1), other reference strains from the United Kingdom (UK) [21], Scotland [25], and Switzerland [31] were used to supplement the Dutch ones. These data sets were identified among other published data sets from New Zealand, Australia, Curaçao, United States of America, and Finland (references available upon request), accessible in PubMLST (http://pubmlst.org/). The data sets from the UK, Scotland, and Switzerland were identified based on the similarity of the *C. jejuni* and *C. coli* ST frequency distributions of human isolates in these countries with those of human isolates in The Netherlands. The Euclidean distance was used as similarity metric in principal component analysis (PCA) [32]. The PCA revealed that the human isolates from The Netherlands were indeed most similar to the human isolates from the UK, Scotland, and Switzerland [33].

**Table 1:** *Campylobacter* strains used to feed the asymmetric island model for source attribution.

Country	Human	Chicken	Cattle	Sheep	Pig	Environment	Reference
The Netherlands	980†	210	9	0	13	106 (water)	[30] and data‡
United Kingdom	0	0	46	72	5	50 (sand)	[21]
Scotland	0	0	90	88	15	133 (wild birds)	[25]
Switzerland	0	0	23	0	100	0	[31]
Total	980	210	168	160	133	289	

† Obtained from the CaSa study [10].

‡ Provided by the Central Veterinary Institute (CVI) in Lelystad, The Netherlands.

For the purposes of this study, the identified reservoir data [21], [25], [31], and those available for The Netherlands (i.e. [30] and additional data supplied by the CVI) were pooled and arranged in five groups: 1) chicken; 2) cattle; 3) sheep; 4) pigs; and 5) the environment (Table 1). Environmental strains were those sourced from water, sand, and wild birds, and were treated as a “reservoir” as well in the attribution analysis. Although the environment cannot be considered as a single amplifying host for *Campylobacter* spp. but only as a “pseudo-reservoir” collecting strains from a variety of different hosts, the STs found in environmental samples have hardly ever been found in other reservoirs [23], [34]. Therefore, the environment was considered as a proxy for other unidentified reservoirs, putatively of primarily wildlife origin [23], [34].

### Attribution analysis

The Asymmetric Island (AI) model [22] was used to attribute human *Campylobacter* infections to the four putative animal reservoirs and to the environment. The AI model is a coalescent-based model derived from a generalization of the Wright's island model. It incorporates a Bayesian approach for modeling the genetic evolution and zoonotic transmission of the *Campylobacter* strains using their allelic profiles, accounting for relatedness among STs. The model estimates the mutation and recombination rates within the reservoirs, as well as the migration rates between reservoirs and from each reservoir to the human population. These migration rates are used to estimate the relative contribution of each reservoir to human infections [22]. By modeling the evolutionary processes of mutation and recombination, the AI model accounts for the occurrence of novel alleles, or novel combinations of alleles, in strains from humans that are unobserved in reservoir populations [22].

For every case, the AI model estimates a relative assignment posterior probability (*Pr*) to originate from each reservoir. The proportion of human infections attributed to a given reservoir is calculated as the sum of its *Pr* over cases divided by the total number of cases.

For each reservoir, differences in *Pr* were tested among age groups, degrees of urbanization, and seasons using the Kruskal-Wallis test (KW); and between genders using the Mann-Whitney U test (MW) (α-level: 0.05).

### Risk factor analysis

We repeated the analysis of risk factors for human campylobacteriosis as previously applied [10], using the 737 cases typed with MLST and the 3119 controls enrolled. *C. jejuni* and *C. coli* infections were analyzed together. For preliminary significance testing, we assessed the association of 131 putative risk factors with *Campylobacter* infection using unconditional logistic regression with the matching variables and the level of education (categorized as: low = primary, lower vocational or lower secondary education; intermediate = intermediate vocational, intermediate secondary or higher secondary education; high = higher vocational and university education) included as covariates, which is the method of choice for frequency-matched data [35]. Factors showing a *p*-value lower than 0.10 for the association with the outcome in the single-variable analysis were selected for inclusion in a multivariable logistic regression model. A backward stepwise selection procedure was applied and variables with a *p*-value lower than 0.05 were retained in the final model. The population attributable risk (PAR) and the population preventable risk (PPR) of each significant factor were calculated based on multivariable odds ratios (OR) and the prevalence of exposure in cases. Similarly, confidence intervals of PARs and PPRs were derived from the confidence intervals of the multivariable ORs [10].

To investigate risk factors for human campylobacteriosis caused by *Campylobacter* strains highly associated with the different reservoirs, we constructed several logistic regression models that included separate subsets of cases assigned to the different reservoirs on the basis of the ranking of their estimated *Pr*s. The assignment of cases to the different reservoirs was performed similarly to previous case-studies [36], [37]. The distribution of *Pr* for each reservoir was assessed and a cut-off point was determined to provide a reasonable balance between the number of cases assigned to each reservoir and the confidence as to their correct assignment derived by the highest possible *Pr*.

**Figure 1:**
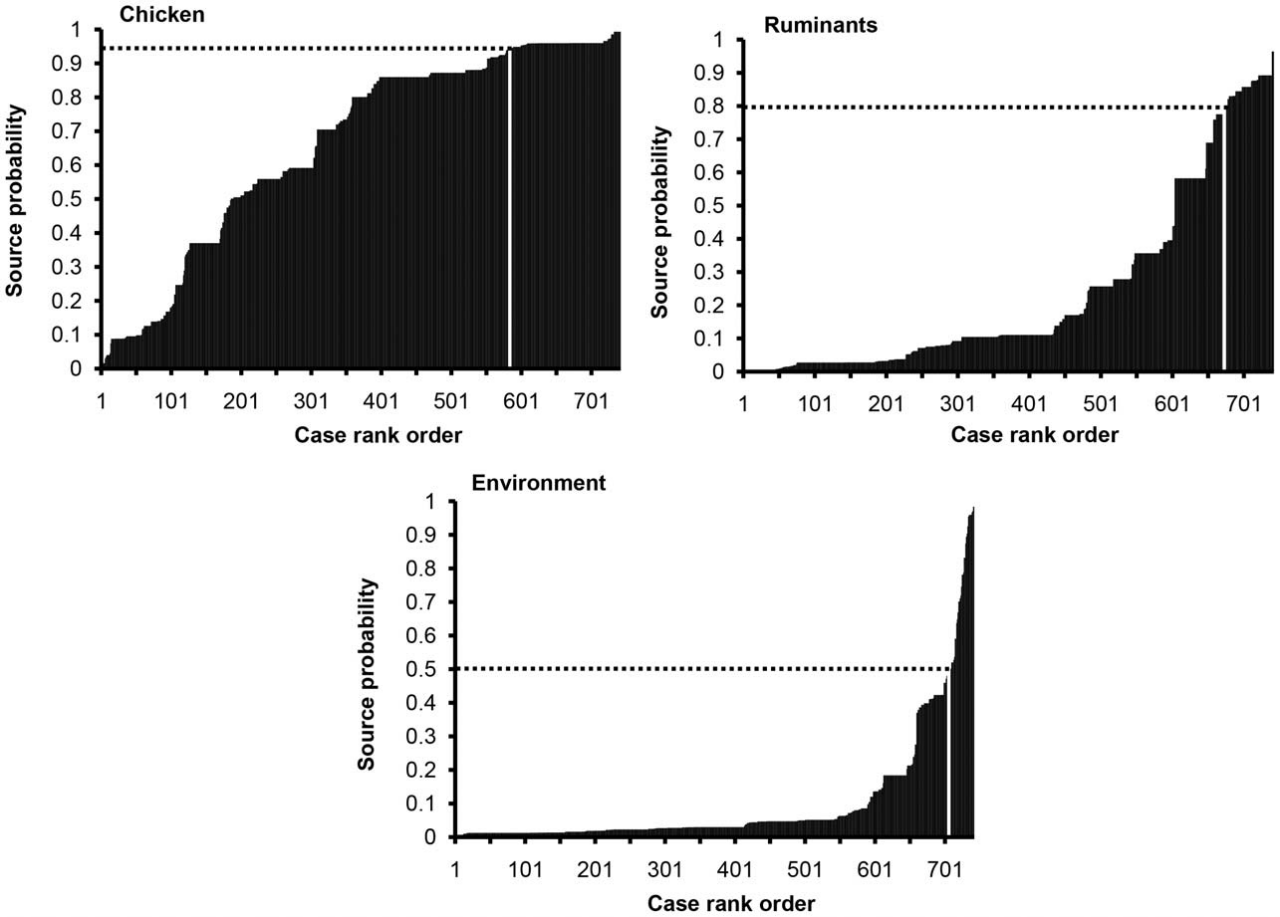
Rank ordered assignment source probability per human case (vertical columns). The white vertical columns indicate the cut-off points beyond which cases were selected for inclusion in the risk factor analysis. Cases are in ascending order according to the source probability to aid visualization.

For infections of probable chicken, ruminant (cattle plus sheep), and environmental origin, separate logistic regression models that included only those cases with at least 50% probability (cut-off: *Pr*≥0.50) of originating from each of these reservoirs were constructed. For infections of probable chicken and ruminant origin, further logistic regression models were constructed for a range of other consecutive cut-off points at regular intervals of 0.05, from *Pr*≥0.50 to *Pr*≥0.95 for chicken, and from *Pr*≥0.50 to *Pr*≥0.80 for ruminants (Figure 1 and Table 2). The low numbers of the remaining cases did not allow for the construction of further models based on successive cut-off points. For the environment, it was only possible to construct a logistic regression model using the cut-off point of *Pr*≥0.50 (Figure 1 and Table 2) because there were too few cases with a higher *Pr* to enable consistent estimation.

**Table 2:** *Campylobacter* sequence types of human cases assigned to chicken, ruminant, and the environment in the risk factor analysis.

Chicken^a^	Ruminants^b^	Environment^c^
44	19	350
227	22	447
230	38	508
290	61	586
353	104	587
354	206	637
400	270	696
443	403	710
584	432	861
606	475	1080
775	658	1539
801	1519	2123
859	2156	2130
875	2288	2151
883		2187
978		3015
1073		3130
1191		4276
1583		4279
1600		4282
1707		4300
1728		4307
1957		4308
2034		4314
2183		
2324		
2553		
2807		
2808		
2844		
2882		
2899		
3016		
4269		
4271		
4280		
4283		
4292		

a) 143 cases, mean *Pr* for chicken = 0.96; range: 0.95–0.99.

b) 67 cases, mean *Pr* for ruminants = 0.87; range: 0.80–0.96.

c) 34 cases, mean for environment *Pr* = 0.76; range: 0.50–0.98.

The final cut-off points represented the best trade-off between the increasing *Pr* for a given reservoir (i.e. increase in reservoir specificity) and the decreasing number of cases includable in the models (i.e. decrease in statistical power and failure of the model to converge). For infections of probable chicken origin, 143 cases with a mean *Pr* for chicken of 0.96 (range: 0.95–0.99) were selected. For infections of probable ruminant origin, 67 cases with a mean *Pr* for ruminant of 0.87 (range: 0.80–0.96) were selected. Finally, for infections of probable environmental origin, 34 cases with a mean *Pr* for environment of 0.76 (range: 0.50–0.98) were selected (Figure 1 and Table 2).

For infections of probable pig and sheep origin, the construction of any regression model was technically possible, yet epidemiologically inappropriate, because there were no or just two cases with *Pr*≥0.50 for sheep and pig, respectively. Moving the cut-off point to a *Pr*<0.50 for sheep and pig would have resulted in the inclusion of many cases nearly equally attributed to the different reservoirs, making the risk factor analysis unclear and relatively uninformative. For the risk factor analysis, cattle and sheep were thus combined into ruminants as done previously [36], [37]. This option appears to be justified by the weak discrimination of *Campylobacter* strains from sheep and cattle when using MLST [14], [22].

To explore if the risk factors of the multivariable logistic regression models differed according to age, sex, degree of urbanization, season, and level of education we also tested the significance of their interactions. The final multivariable logistic regression models were therefore expanded to include significant interaction terms.

For simplicity, only the results of the final multivariable regression models based on the aforementioned final cut-off points were presented. Although food and non-food related risk factors were estimated together, they were presented separately to improve readability of the tables. All regression models maximized to the *Pr* for a given reservoir showed an overall statistical significance (likelihood ratio χ^2^ test, *p*<0.05) and an acceptable goodness-of-fit (Hosmer-Lemeshow test, *p*>0.05).

For all risk factor analyses, the controls were used as common comparison group. The matching variables and the level of education were always included as covariates in all regression models to control for confounding, as the *Pr*-based selection of cases slightly skewed them from the controls with respect to these confounders. To support the accuracy of inferences of regression models with <5 cases per variable, bias-corrected bootstrap confidence intervals were also calculated (1000 replications) and compared with the standard ones [38]. As these confidence intervals did not differ significantly, the standard ones were reported. Statistical analyses were performed using STATA 11.2.

## Results

### Human multilocus sequence types and clonal complexes

**Figure 2:**
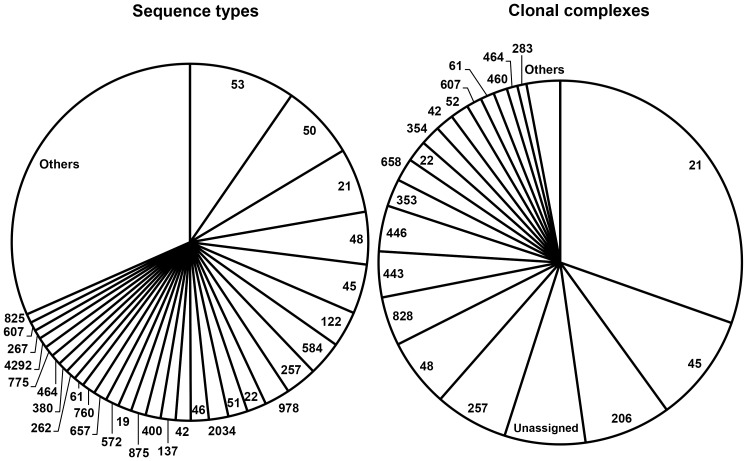
Human *Campylobacter* strains per clonal complex and sequence type assigned with MLST. The category ‘others’ includes clonal complexes and sequence types with less than five isolates.

**Figure 3:**
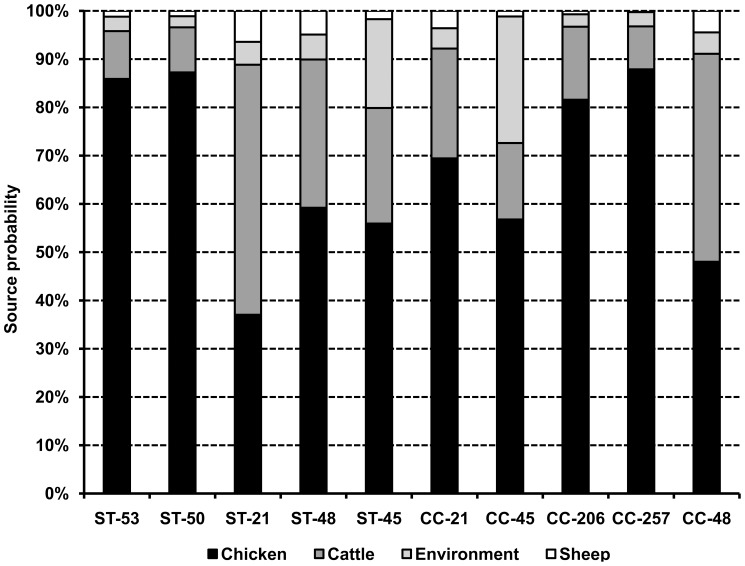
Attributed probability (%) for the five most represented sequence types and clonal complexes to originate from chicken, cattle, sheep, and the environment. The probability for pigs is not viewable because it is <1%.

Overall, the 737 *Campylobacter* strains were assigned to 154 STs belonging to 28 CCs. Twenty-eight STs were unassigned to a previously identified CC. The frequency of genotypes was highly skewed, with ST-53, ST-50, ST-21, ST-48, and ST-45 accounting for more than a quarter of all isolates, and CC-21, CC-45, CC-206, CC-257, and CC-48 accounting for more than half of all isolates (Figure 2). The attribution analysis revealed that ST-50, ST-53, ST-48, and ST-45 were predominantly related to chicken, with a substantial contribution from cattle in ST-48 and ST-45 (Figure 3). ST-21 was mostly related to cattle and chicken (Figure 3). Chicken was also the predominant source for isolates belonging to CC-257, CC-206, CC-21, CC-45, and CC-48, but a substantial contribution from cattle was also found in CC-48 and CC-21, and from the environment in CC-45 (Figure 3).

For *C. jejuni*, the frequencies of STs and CCs followed the same ranking as the aforementioned ones; whereas for *C. coli*, the five most frequent STs were ST-825, ST-827, ST-1614, ST-854, and ST-1600, all belonging to CC-828, which accounted for 68% of *C. coli* isolates and was predominantly related to chicken (*Pr* = 0.69) and cattle (*Pr* = 0.16).

Significant age relationships were found for ST-53 (χ^2^ test, *p*<0.001) and its CC, CC-21 (χ^2^ test, *p* = 0.002). The highest relative frequency of ST-53 was found in children and adolescents (0–4 and 5–17 years, which accounted together for 48% of ST-53 isolates), whereas that of CC-21 (24% of CC-21 isolates) was found in young adults (18–29 years). ST-21 was significantly over-represented in urbanized areas (53%; χ^2^ test, *p* = 0.036). Significant seasonal effects were found for ST-48 (χ^2^ test, *p* = 0.023) and its CC, CC-48 (χ^2^ test, *p* = 0.028), which showed the lowest relative frequencies in the spring (3% and 4%, respectively), peaked in the summer (43% and 40%, respectively) and had intermediate frequencies (23–31%) during autumn-winter months.

### Attribution of human infections

Overall, the AI model estimated that the majority of human infections (489; 66.2%) originated from chicken, followed by cattle (153; 20.7%), environment (74; 10.1%), sheep (19; 2.5%), and pigs (2; 0.3%).

**Figure 4:**
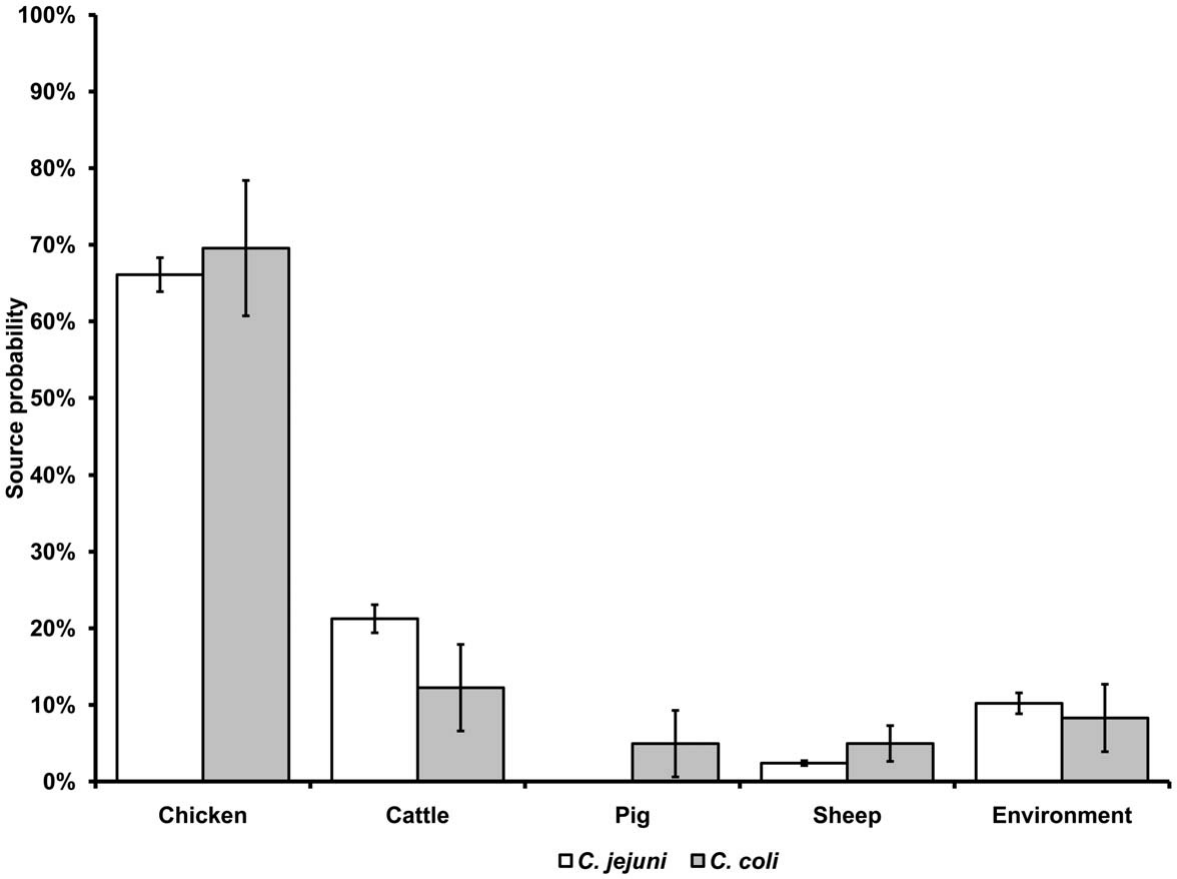
Overall mean probability (%) and 95% confidence interval for human *C. jejuni* (*n* = 696) and *C. coli* (*n* = 41) infections to originate from chicken, cattle, pig, sheep, and the environment.

The 696 *C. jejuni* cases were attributed as follows: chicken, 66.1% (460 cases); cattle, 21.2% (148); environment, 10.2% (71); sheep, 2.4% (17); and pigs, 0.01% (<1) (Figure 4). The 41 *C. coli* cases were attributed as follows: chicken, 69.6% (29 cases); cattle, 12.2% (5); environment, 8.9% (3); sheep, 5.0% (2); pigs, 4.9% (2) (Figure 4).

The *Pr*s for cattle and pig were significantly different between *C. jejuni* and *C. coli* (cattle: MN, *p* = 0.001; pig: MN, *p*<0.001). Significant effects of urbanization were found for the chicken reservoir (KW, *p* = 0.023), which showed the highest median *Pr* (0.84) in cases from urbanized areas. A significantly (MN, *p* = 0.018) higher *Pr* for chicken was also found for young children aged 0–4 years living in urban areas (median *Pr*: 0.86; *n* = 15) compared with those living in rural areas (median *Pr*: 0.59; *n* = 33). A significant seasonal effect (KW, *p* = 0.020) was found in *Pr* for the environmental reservoir, which peaked in the spring followed by a trough in the summer and autumn, with a small peak in the winter.

### General risk factors for campylobacteriosis

In contrast to the previous study [10], we used a smaller sample of cases and *C. jejuni* and *C. coli* were analyzed together. Nevertheless, the direction and strength of the factors associated with campylobacteriosis in the final multivariable model (Table 3 and Table 4) were comparable with the previous results [10]. With a PAR of 28%, consumption of chicken was the most important risk factor, followed by consumption of barbecued (18%) and undercooked (16%) meats, and eating in a restaurant (11%) (Table 3). However, a significantly higher risk was observed only when barbecued meat was consumed by patients living in non-urban areas (Table 3).Of the non-food risk factors (Table 4), strong associations were found for recent use of proton-pump inhibitors (22%), and having a chronic gastrointestinal disease (20%).

**Table 3:** Multivariable odds ratios and percent PAR or PPR (and 95% confidence intervals) for food-related risk factors for human campylobacteriosis according to the attributed origin of the *Campylobacter* strain (chicken, ruminant, and the environment).

Risk factor (% imputed missing values*)	Overall^a^	Chicken^b^	Ruminants^c^	Environment^d^
*Food consumption*				
Chicken (1)	**1.5 (1.2–1.9)**	**1.9 (1.2–2.9)**	ns	ns
	**28% (13–41%)**	**42% (14–60%)**		
Beef (1)	ns	0.6 (0.4–0.9)	ns	ns
		30% (7–44%)		
Pork (2)	ns	0.7 (0.5–0.9)	ns	ns
		16% (5–26%)		
Tripe (1)	ns	ns	**4.0 (1.1–14.2)**	ns
			**12% (1–37%)**	
Game (0)	ns	ns	ns	**3.3 (1.4–7.8)**
				**37% (10–64%)**
Undercooked meat (5)	**2.1 (1.6–2.7)**	ns	ns	ns
	**16% (10–23%)**			
Barbecued, grilled, or microwaved meat (5)	**18% (10–25%)**	ns	**63% (41–78%)**	ns
in urban areas	**1.2 (0.7–2.2)^ns^**	ns	0.8 (0.1–7.3)^ns^	ns
in urbanized areas	**1.7 (1.3–2.2)**	ns	**7.1 (3.2–15.6)**	ns
in rural areas	**3.0 (1.8–4.9)**	ns	**4.1 (1.3–14.2)**	ns
Meat in paste (croquette, meat roll, pastry) (5)	0.8 (0.6–1.0)	ns	0.5 (0.2–0.8)	ns
	8% (0–13%)		14% (1–22%)	
Pasteurized milk (1)	0.8 (0.6–0.9)	ns	ns	ns
	15% (6–29%)			
Pasteurized dairy other than milk or cheese (2)	0.6 (0.5–0.7)	0.4 (0.3–0.7)	0.5 (0.3–0.9)	0.3 (0.1–0.8)
	34% (25–42%)	41% (20–48%)	37% (7–51%)	48% (13–61%)
Stir-fried vegetables (3)	ns	ns	ns	0.3 (0.1–0.9)
				11% (2–16%)
Salad (2)	0.7 (0.6–0.9)	ns	0.4 (0.2–0.9)	ns
	7% (2–9%)		10% (2–13%)	
Fruit with peel (2)	0.7 (0.6–0.8)	ns	ns	ns
	13% (9–17%)			
Chocolate (2)	0.6 (0.5–0.7)	0.5 (0.4–0.7)	ns	ns
	22% (17–28%)	22% (17–34%)		
Nuts (3)	0.6 (0.5–0.8)	ns	0.5 (0.3–0.9)	ns
	13% (6–16%)		16% (0–22%)	
Seafood (4)	0.5 (0.4–0.7)	0.6 (0.4–0.9)	ns	ns
	14% (8–16%)	13% (3–20%)		
*Eating habits*				
Eating in a restaurant (0)	**1.3 (1.1–1.6)**	ns	ns	ns
	**11% (4–20%)**			
Vegetarian diet (0)	0.4 (0.2–0.9)	ns	ns	ns
	1% (0–1%)			
Eating chicken once a month or less (3)	ns	ns	**1.7 (1.0–2.9)**	ns
			**25% (1–47%)**	
*Kitchen hygiene*				
Not cleaning a knife when using it for raw meat and other foods (1)	**1.7 (1.1–2.6)**	ns	ns	ns
	**4% (6–9%)**			
Washing hands before food preparation (0)	0.6 (0.4–0.9)	ns	ns	ns
	1% (0–2%)			

Multivariable odds ratios are also adjusted for age, sex, degree of urbanization, season, and level of education. PAR (population attributable risk) and PPR (population preventable risk) are based on the multivariable odds ratios. Risk factors are in bold, protective factors in normal font.

ns = not significant (*p*>0.05).

* Fraction of imputed missing values in the whole dataset.

a) 737 cases; mean *Pr* for chicken = 0.66 (range: 0.00–0.99); mean *Pr* for ruminants = 0.23 (range: 0.00–0.96); mean *Pr* for environment = 0.10 (range: 0.00–0.98).

b) 143 cases; mean *Pr* for chicken = 0.96; range: 0.95–0.99.

c) 67 cases; mean *Pr* for ruminants = 0.87; range: 0.80–0.96.

d) 34 cases; mean *Pr* for environment = 0.76; range: 0.50–0.98.

**Table 4:** Multivariable odds ratios and percent PAR or PPR (and 95% confidence intervals) for non-food related risk factors for human campylobacteriosis according to the attributed origin of the *Campylobacter* strain (chicken, ruminant, and the environment).

Risk factor (% imputed missing values*)	Overall^a^	Chicken^b^	Ruminants^c^	Environment^d^
*Contact with animals*				
Contact with dog(s) owned by other people (3)	0.6 (0.5–0.8)	ns	ns	ns
	8% (4–10%)			
Contact with pets and/or farm animals outside the household (1)	ns	ns	ns	0.4 (0.2–1.0)
				17% (1–22%)
Ownership of several dogs, at least one dog <1 year-old (0)	**2.5 (1.1–5.8)**	ns	ns	ns
	**2% (1–7%)**			
Ownership of several dogs, all dogs >1 year-old (0)	ns	ns	ns	**3.5 (1.0–12.0)**
				**33% (1–54%)**
Ownership of cat(s) (1)	**1.4 (1.2–1.8)**	ns	ns	ns
	**10% (5–17%)**			
*Recent use of medication*				
Antibiotics (0)	0.4 (0.2–0.8)	ns	ns	ns
	1% (0–2%)			
Proton-pump inhibitors (0)	**3.7 (2.5–5.5)**	**4.7 (2.4–9.1)**	**5.7 (2.2–16.15.3)**	ns
	**22% (14–33%)**	**34% (11–53%)**	**34% (11–58%)**	
*Other*				
Swimming in a domestic swimming pool (0)	ns	ns	ns	**28% (2–64%)**
in the spring season	ns	ns	ns	**16.8 (2.6–107.6)**
in the summer, winter or autumn seasons	ns	ns	ns	**2.5 (0.4–14.4)^ns^**
Contact with people with gastroenteritis symptoms outside the household (3)	**1.5 (1.1–2.1)**	**1.8 (1.1–3.0)**	ns	**3.4 (1.3–8.7)**
	**6% (1–12%)**	**10% (1–23%)**		**35% (6–63%)**
Having a chronic gastrointestinal disease (0)§	**2.4 (1.8–3.2)**	**1.8 (1.1–3.1)**	ns	**5.0 (2.1–12.1)**
	**20% (13–28%)**	**12% (2–27%)**		**50% (22–74%)**
Occupational exposure to animals (0)	ns	ns	**3.2 (1.2–9.0)**	ns
			**17% (2–41%)**	

Multivariable odds ratios are also adjusted for age, sex, degree of urbanization, season, and level of education. PAR (population attributable risk) and PPR (population preventable risk) are based on the multivariable odds ratios. Risk factors are in bold, protective factors in normal font.

ns = not significant (*p*>0.05).

* Fraction of imputed missing values in the whole dataset.

a) 737 cases; mean *Pr* for chicken = 0.66 (range: 0.00–0.99); mean *Pr* for ruminants = 0.23 (range: 0.00–0.96); mean *Pr* for environment = 0.10 (range: 0.00–0.98).

b) 143 cases; mean *Pr* for chicken = 0.96; range: 0.95–0.99.

c) 67 cases; mean *Pr* for ruminants = 0.87; range: 0.80–0.96.

d) 34 cases; mean *Pr* for environment = 0.76; range: 0.50–0.98.

§ Includes Crohn's disease, irritable bowel disease (IBD), irritable bowel syndrome (IBS), or celiac disease.

With a PPR of 34%, consumption of pasteurized dairy products other than milk and cheese (i.e. mostly yoghurt) was the most important protective factor, followed by consumption of chocolate (22%), pasteurized milk (15%), seafood (14%), fruit (13%), nuts (13%), meat in paste (8%), and salad (7%) (Table 3). Of the non-food protective factors, contact with dogs owned by other people was the most important one (8%) (Table 4).

### Risk factors for chicken-associated campylobacteriosis

For chicken-associated campylobacteriosis, consumption of chicken was the most important risk factor (PAR 42%) (Table 3), followed by recent use of proton-pump inhibitors (34%), having a chronic gastrointestinal disease (12%), and contact with people with gastroenteritis symptoms outside the household (10%) (Table 4). Important protective factors were consumption of yoghurt (PPR 41%), beef (30%), pork (16%), and seafood (13%) (Table 3).

### Risk factors for ruminant-associated campylobacteriosis

With a PAR of 63%, consumption of barbecued meat was the most important risk factor for ruminant-associated campylobacteriosis (Table 3). However, the risk posed by the consumption of barbecued meat was significantly higher only for patients living in non-urban areas. Other important risk factors were: consumption of tripe (12%), eating chicken rarely, i.e. once a month or less (25%) (Table 3), recent use of proton-pump inhibitors (34%), and occupational exposure to animals (17%) (Table 4). Important protective factors were consumption of yoghurt (PPR 37%), nuts (16%), and meat in paste (14%) (Table 3).

### Risk factors for environment-associated campylobacteriosis

Consumption of game was the only food-related risk factor for campylobacteriosis of probable environmental origin (PAR 37%) (Table 3). Other important risk factors were: having a chronic gastrointestinal disease (50%), contact with people with gastroenteritis symptoms outside the household (35%), swimming in a domestic swimming pool (28%), and ownership of several adult dogs (33%) (Table 4). However, a significantly higher risk was observed only when patients swam in a domestic swimming pool during the spring (April–June), but not during the other seasons (the risk of swimming during the summer, autumn, and winter months was equally insignificant; thus, these strata were combined, Table 4).

Important protective factors were consumption of stir-fried vegetables (PPR 11%) (Table 3) and contact with pets and/or farm animals outside the household (17%) (Table 4).

## Discussion

This is the first case-control study in which risk factors at the point of exposure for human campylobacteriosis are investigated in relation to the attributed reservoirs based on MLST profiles. Previous studies [36], [37] examined risk factors for reservoir-associated campylobacteriosis in a similar, albeit more limited, way, as only a small number of risk factors about demographic characteristics (e.g. age, sex, resident location, etc.) were investigated and a case-case approach was used.

### *Campylobacter* multilocus genotypes

*Campylobacter* populations are regarded as genetically highly diverse, even when considering their core-genome using MLST [9]. With 154 STs identified among 737 human cases, our results indicate that considerable variety exists also in the Dutch *Campylobacter* population. Rare STs were also considerably represented, as STs occurring once accounted for 46% of all STs. Besides this large variety, there was some evidence indicating that certain genotypes can emerge and predominate in specific age groups, areas, and seasons, although most of the commonest genotypes were broadly distributed and recurrent over time.

The main STs and CCs identified here have been reported worldwide [21], [23], [25], [39]–[44] and were typical of previous reports from The Netherlands [20], [30]. Most of these studies, however, were geographically and temporally limited; thus, the extent to which the predominant genotypes, both in humans and reservoirs, correspond to stable geographical structuring or to a transient expansion could not be investigated.

In our study, CC-21 was the most represented CC and was predominated (32%) by ST-53, which was also the most common ST in the whole data set. Although both CC-21 and ST-53 were primarily attributed to chicken, they were over-represented among cattle isolates in Scotland [25] and Finland [40], and among cattle and chicken isolates in the UK [21] and multi-country collections [43]. To a lesser extent, there is also evidence for sheep and environment to be involved [21], [22], [25], [40], [43], [44]. This also applies to the ubiquitously sourced ST-50, ST-21, ST-48, and ST-45, although they seem to be predominant in chicken (ST-50 and ST-45) and cattle (ST-21 and ST-48) [21], [23], [40], [43]. Differences in host preference among *Campylobacter* genotypes observed in this study may be due to niche adaptation, geographic separation, host-related factors (e.g. immunity, behaviors with respect to potential exposures, etc.), or barriers to genetic exchange [40].

### Attributed reservoirs of human campylobacteriosis

Chicken was estimated to be the most important reservoir of human campylobacteriosis in The Netherlands, accounting for approximately 66% of infections. This is in line with other studies conducted in industrialized countries using the AI model [22]–[24]. The proportion of cases attributable to chicken, however, varied considerably among these studies (56% [22] and 76% [23], 24]). Besides variations in local epidemiology, such divergences are mostly due to the consideration of different reservoirs, which may affect the proportions of attributed infections. For instance, Mullner et al. [23] did not consider pigs; Sheppard et al. [24] kept wild birds, environment, and turkey as separated sources; Wilson et al. [22] included also rabbit and kept wild birds, sand, and water separated.

We found that *Pr*s for pig and cattle were significantly different between *C. jejuni* and *C. coli* strains. A higher *Pr* for cattle was found in *C. jejuni* compared with *C. coli*, whereas a higher *Pr* for pig was found in *C. coli* compared with *C. jejuni*. This supports evidence indicating that *C. jejuni* is more prevalent than *C. coli* in cattle and that the inverse situation holds for pigs [9]. We also found that chicken was the major reservoir for campylobacteriosis in young children living in urban areas compared with their rural counterparts, for which cattle seemed to be more important, although the difference for cattle was not clearly significant (data not shown). The same finding was previously observed in Scotland [25] and New Zealand's North Island [37], supporting the hypothesis that the main source of campylobacteriosis for young children depends on residence location: chicken (consumption) is a more important source of infection in urban dwellers, while infection from cattle seems to be more likely to occur in rural areas, possibly via environmental pathways [25]. In The Netherlands, cattle density has also been associated with an increased risk for Shiga toxin-producing *Escherichia coli* (STEC) O157 infection in young children living in rural areas [45]. Together these results suggest that the risk of encountering and becoming diseased with enteropathogens putatively shed by cattle is considerable in young children living in rural areas.

A significant seasonal effect was found for the environmental reservoir. *Campylobacter* is widespread in the environment where it generally gives clues to recent fecal contamination, agricultural run-off, and sewage effluent [46]. Although intestinal carriage of *Campylobacter* is ubiquitous in animals, the environmental contamination varies seasonally depending on factors such as stress, changes in diet, and indoor/outdoor housing of animals [46]. The significant seasonal pattern of campylobacteriosis of probable environmental origin may reflect both the year-round variation in *Campylobacter* die-off rates in varying environments and the increased propensity of people for outdoor recreational activities, especially water activities, during the warm season, which may entail transmission from outdoor-reared animals and so far unidentified wildlife reservoirs.

### Reservoir-specific risk factors for campylobacteriosis

While the attribution analysis quantified the relative contributions of the considered reservoirs to human infections, the risk factor analysis identified the excess risks for infections that were highly associated with these reservoirs, allowing for the identification of the possible pathways by which *Campylobacter* infection may be acquired from a given reservoir, as well as their quantification in terms of PAR. For instance, only up to 42% (14–60%) of the highly chicken-associated infections could be ascribed to consumption of chicken, supporting the hypothesis that a considerable part of infections originating from chicken is acquired by pathways other than food, such as the environment [18], or by cross-contamination to commodities, utensils, and foods other than chicken [47]. Indeed, it has been suggested that sporadic campylobacteriosis is more likely to occur because of cross-contamination from raw poultry products than because of consumption per se [12].

Some factors may be significantly associated with infections attributed to a given reservoir just because these infections have a residual contribution from reservoirs other than those to which they were attributed. Although the selection of cases for the risk factor analysis was based on the highest possible *Pr*s, residual attributions were 4%, 13%, and 24% in chicken-, ruminant-, and environment-associated infections, respectively. Nevertheless, all risk factors were associated in an epidemiologically plausible way according to the reservoir in question. For instance, consumption of chicken was a risk factor for infections of chicken origin whereas the consumption of beef and pork appeared to protect against chicken-associated infections. Plausibly, a person may be “protected” against infection with the most chicken-associated *Campylobacter* strains when exposed to reservoirs other than chicken, such as pig and cattle. Furthermore, consumption of tripe, barbecued meat, and seldom or never consumption of chicken were risk factors for infections attributed to ruminants. Possibly, people consuming chicken rarely may consume meats and other edible products from ruminants more frequently. Although we did not have any information about the type of meat cooked at the barbecue, it is clear that red meats are more likely to be consumed rare when barbecued, and thus more likely to harbor viable *Campylobacter* due to incomplete cooking. Besides undercooking, barbecuing usually provides many opportunities for re- and cross-contamination. The fact that the risk posed by barbecued meat was higher in patients living in non-urban areas, and insignificant in those living in urban ones, is supportive of the aforementioned hypothesis that ruminant-associated infections are more likely to occur in the countryside [25], [37]. Working with animals was also a risk factor for infections attributed to ruminants, supporting another hypothesis stating that these infections may be acquired, to a considerable extent, through animal contact rather than food [23], [24].

Consumption of game and swimming in a domestic swimming pool increased the risk for infections of probable environmental origin. In our study, the environmental reservoir included strains from wild birds, water, and sand. Although water and sand cannot be considered as amplifying hosts, they can act as vehicles delivering an exposure possibly from primary wildlife reservoirs [23], [34]. In The Netherlands, *Campylobacter* is commonly found in recreational water [48] and domestic swimming pools mainly consist of temporary outdoor inflatable swimming pools of limited capacity, which can easily become contaminated by bird feces. Moreover, it is likely for cleaning and maintenance procedures of swimming pools (e.g. water chlorination) to be less strictly applied in a domestic context. However, we found that the risk posed by domestic swimming pools was only significant in the spring but not in the other seasons. This is in accordance with our other finding indicating that the importance of the environmental reservoir varies seasonally, with a major peak in the spring. While most (outdoor) swimming pools are unusable during autumn-winter months, several British studies (reviewed by Jones [46]) have evidenced that there is a negative correlation between hours of sunshine and *Campylobacter* presence in recreational water, with significantly lower isolation rates in the summer compared to the other seasons corresponding to elevated ultraviolet radiation levels and higher temperatures, two conditions that greatly affect the survival of *Campylobacter* spp. outside the host. Moreover, it is possible that swimming pools are cleaned more frequently in the summer as a result of their more frequent use, or that other exposures and reservoirs play competitively a more prominent role in the summer.

We found that recent use of proton-pump inhibitors, having a chronic gastrointestinal disease, and contact with people with gastroenteritis symptoms outside the household were risk factors for infections attributed to different reservoirs. It is conceivable that the neutralizing effect of proton-pump inhibitors on gastric acidity may enhance *Campylobacter* survival during its passage through the stomach and that a disturbed intestinal function may facilitate infection [10]. However, it is also possible that *Campylobacter* infections are more likely to be diagnosed in people affected by chronic gastrointestinal diseases, as these people may be under more frequent medical attention (closer surveillance) and diagnostic thoroughness. Person-to-person transmission is uncommon for campylobacteriosis but it probably occurs with no particular preference for the primary reservoir of the *Campylobacter* strain involved. However, it is worth mentioning that this risk was particularly pronounced for *Campylobacter* strains of probable environmental origin. Considering that our environmental strains were sourced from water and sand among others, and that person-to-person transmission seems particularly important in children [10], it can be speculated that sand (particularly the one in playground sand-boxes) and recreational water can act as a vehicle for transmission among humans as well.

Consumption of several non-meat foods, including fruits, vegetables, dairy (mostly yoghurt), and seafood, were protective against infections attributed to different reservoirs. It is believed that these foods may have genuinely beneficial effects on general health by inhibiting bacterial growth, enhancing general immunity to infection, and altering the intestinal flora in a way that prevents infection [10], [12], [13], [15].

### Limitations and possible sources of bias

We supplemented Dutch data of reservoirs with data from other countries, an approach that has previously been applied [14], [24], but could introduce bias in the attribution estimates. However, it has been shown that the association of multilocus genotypes with specific hosts transcends geographical variations [49]. Therefore, although greater accuracy of attribution estimates is possible with reference data closely sampled in space and time, these are not essential, and reference data from other regions can be used where local data are not available. To address this, we performed a PCA on human data from different countries to identify the corresponding reservoir data that were expected to be close to those present in The Netherlands in 2002–2003 [32]. The underlying assumption was that, if the ST frequency distribution of the human population of The Netherlands resembles that of the human population from another study, then the Dutch reservoir data may well resemble the reservoir data from that study. Apart from the reservoirs and their ST frequency distributions, consumption patterns and exposure pathways were assumed to be comparable, an assumption that has some plausibility among northern European countries. A detailed description of the results of the PCA will be provided in another manuscript that is in preparation.

This study was restricted to *C. jejuni* and *C. coli*. These two species, however, account for up to 98% of infections characterized at species level in The Netherlands [10]; thus, the impact of the other species on attribution estimates was expected to be minimal. It is clear that when exposures are aggregated for *C. jejuni* and *C. coli* infections, the contribution of risk factors primarily associated with *C. coli* may be masked by the numerical superiority of *C. jejuni*. However, cases were split according to *Pr*, and *C. jejuni* and *C. coli* could potentially originate from a same reservoir. The primary outcome of interest was thus to explore reservoir-specific risk factors for campylobacteriosis rather than accounting for *Campylobacter* species-specific risk exposure characteristics. Another limitation concerns the residual contribution to *Pr* by reservoirs other than those to which infections were attributed. To address this in the risk factor analysis, we constructed regression models (when not limited by sample size) that were restricted to subsets of cases with the highest possible *Pr* for each reservoir. The residual contribution, although minimized, creates “noise” which could have masked or diluted some associations, or led to some additional associations, in the risk factor analysis. The latter option could be the case of the ownership of several adult dogs as a risk factor for environment-associated strains. Nonetheless, dogs are often tested positive for *Campylobacter* spp. and it has been suggested that dogs housed in group have a higher prevalence, possibly due to dog-to-dog transmission [50]. Moreover, dog owners may be particularly exposed to *Campylobacter* strains of environmental origin while walking their dogs, and adult dogs living in a group are also more likely to have (unsupervised) outdoor access and can therefore act as a vehicle for *Campylobacter* strains of environmental origin, possibly acquired upon ingestion of contaminated water, predation, necrophagy, and coprophagy. While dog ownership increases the risk for environment-associated infections, contacting an animal outside the household appears to be protective. We speculated that contacting animals other than their own encourages individuals to undertake protective actions, such as hand washing.

Many isolates from the cases included in the previous case-control study [10] were no longer viable and could not be cultured and typed with MLST for the purposes of this study. This could be due to underlying differences in survival among the different *Campylobacter* strains. However, we were able to replicate the results of the previous study [10], suggesting that our subset of cases was not biased and that the non-typed isolates were missed at random.

It has been postulated that repeated exposure to different *Campylobacter* strains may lead to sufficient immunity to provide protection against (severe) clinical illness [1], [51]. In case-control studies, this protective immunity would lead to misclassification, as some controls could have been infected with *Campylobacter* spp. asymptomatically. As cases were identified by passive surveillance, they were likely to represent the most severe, symptomatic infections that occurred in the population. Thus, the identified risk factors especially represent risk factors for severe campylobacteriosis. Other concerns in case-control studies are recall and selection bias. Specifically in this study the recall period for cases was longer than for controls, and controls returning the postal questionnaire could be particularly motivated people with a generally healthier lifestyle, a fact that provides an alternative explanation of why, for example, eating fruits and vegetables were protective factors. Nevertheless, similarly to the previous study [10], these possible biases were explored by conducting multiple imputation checks and case-case analyses (data not shown), which revealed that both recall and selection bias had limited impact on our results.

## Conclusions

A number of case-control studies have explored risk factors for *Campylobacter* infection while other studies have used MLST data to attribute *Campylobacter* infections to animal or environmental reservoirs, as well as used a case-case approach to characterize the risk of becoming infected with *Campylobacter* strains of different origins. Our study attempts to bridge this gap by exploring risk factors at the point of exposure for campylobacteriosis of different origins, using a combined case-control and source attribution analysis.

Our results lend weight to the suggestion that human campylobacteriosis in The Netherlands could greatly be reduced by focusing interventions on chicken and cattle. Chicken seems to be the major reservoir of campylobacteriosis for people living in cities, whereas cattle seems to be more important in their rural counterparts. The importance of the chicken and cattle reservoirs, however, was only partially consistent with food-borne transmission, as alternative pathways, such as direct contact and environmental contamination, do play a role as well, particularly for infections attributed to ruminants.

This study showed that risk factors for *Campylobacter* infection depend upon the attributed reservoir and that the exposure may plausibly direct to the original reservoir when considering those *Campylobacter* strains that are indeed highly associated with the reservoir in question. Combining epidemiological and genotype-based source attribution data was helpful in enhancing risk factor identification and characterization for human campylobacteriosis and in providing a valuable approach for supporting and generating hypotheses. In a broader perspective, our results also indicate that the general concept of genotype-based source attribution modeling for campylobacteriosis makes sense epidemiologically. More Dutch reference strains from other animal reservoirs, such as dogs and cats, as well as different categorizations of food-producing animals, will provide a better discrimination of *Campylobacter* reservoirs and possibly stimulate novel epidemiological insights towards reservoir-specific risk factors and transmission pathways for human campylobacteriosis.
